# Supplementary material for: CRISPR-Cas9 correction of a nonsense mutation in LCA5 rescues lebercilin expression and localization in human retinal organoids
Source: Mol Ther Methods Clin Dev. 2023 May 17;29:522–31. doi: 10.1016/j.omtm.2023.05.012 (PMC10250556; doi:10.1016/j.omtm.2023.05.012)
Supplement: Document S1. Figures S1 and S2 and Tables S1 and S2 [file mmc1.pdf]

OMTM, Volume *29*

Supplemental information

CRISPR-Cas9 correction of a nonsense mutation in *LCA5* rescues lebercilin expression and localization in human retinal organoids

Tess A.V. Afanasyeva, Dimitra Athanasiou, Pedro R.L. Perdigao, Kae R. Whiting, Lonneke Duijkers, Galuh D.N. Astuti, Jean Bennett, Alejandro Garanto, Jacqueline van der Spuy, Ronald Roepman, Michael E. Cheetham, and Rob W.J. Collin

SUPPLEMENTAL INFORMATION

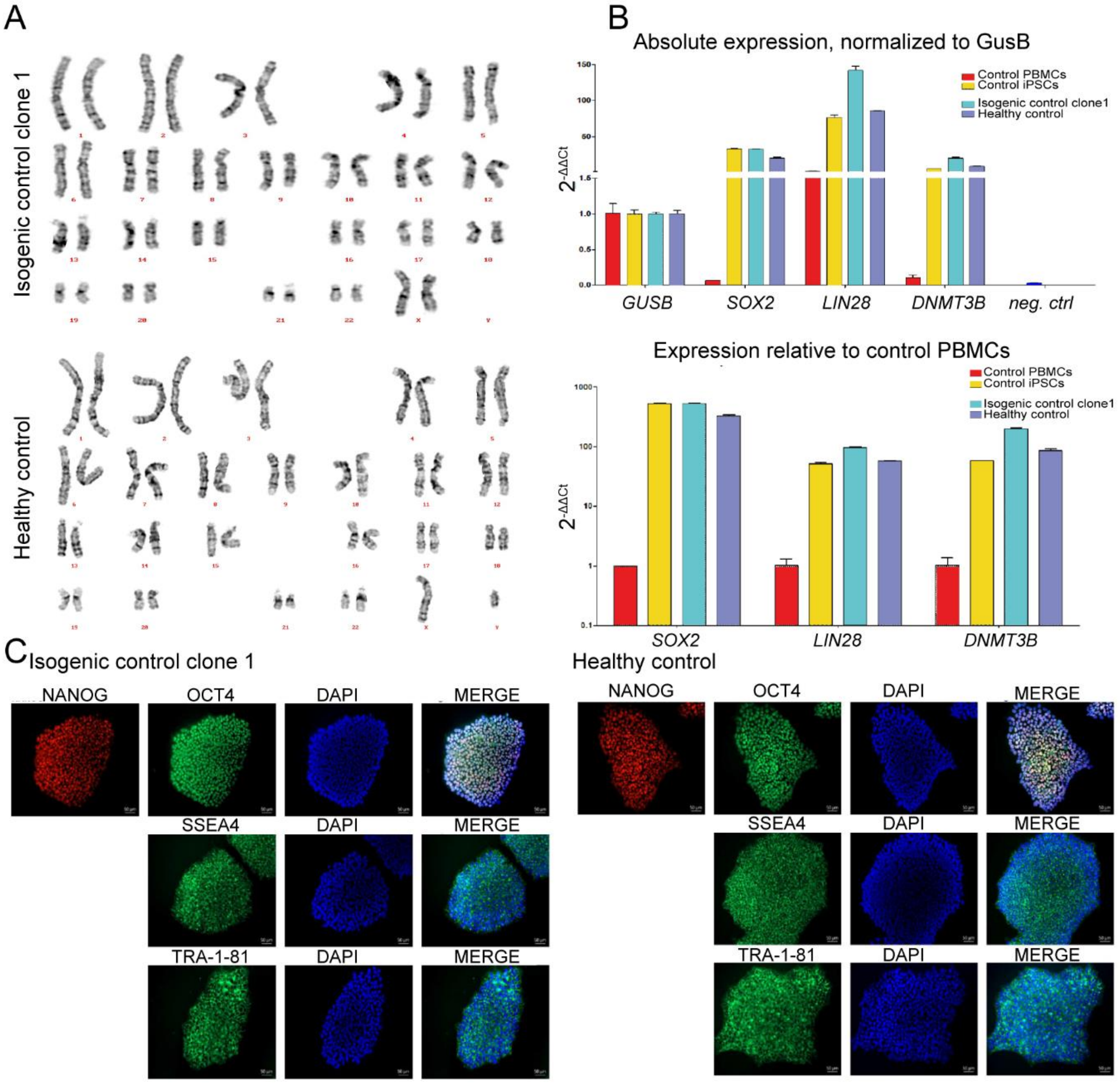

Figure S1. Quality control of healthy control and isogenic control iPSCs. A) Representative karyotypes of isogenic control clone1 and healthy control lines. B) Expression of pluripotency genes SOX2, LIN28, and DNMT3B. C) Immunocytochemical detection of the pluripotency markers OCT4, NANOG, SSEA4, and TRA1-81. Scale bars correspond to 50 µm.

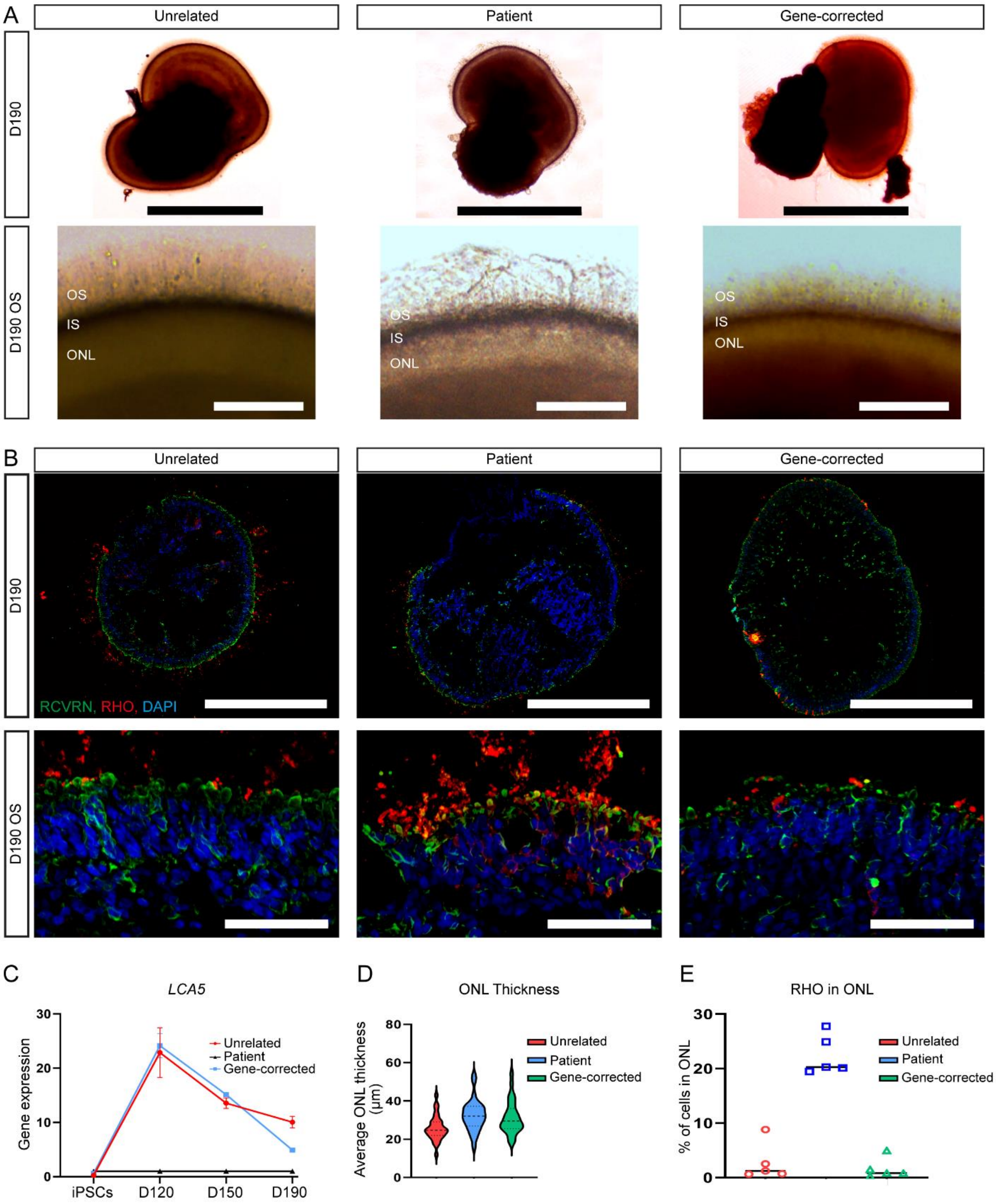

Figure S2. Patient, gene-corrected, and unrelated control human ROs show outer segment-like structures and photoreceptor-cell marker positive cells in the outer nuclear layer. A) D190 ROs generated from LCA5 c.835C>T, gene-corrected and unrelated controls show lamination (top panel, scale bars 1 mm) and brush-like immature outer segments (bottom panel, scale bars 50 µm). B) Representative images of rhodopsin and recoverin-positive outer nuclear layer (ONL) in D190 retinal organoids (top panel scale bars 500 µm and 50 µm, top and bottom panels, respectively). RCVRN

in green, RHO in red, DAPI in blue. C) qPCR of LCA5 expression in iPSCs, D120, D150, and D190 ROs (n=3 ROs per measurement, in duplicate). D) Thickness of ONL among the D190 organoids was 25.8 µm (SD = 6.4 µm), 32.4 µm (SD = 7.9 µm), and 31.2 (SD = 8.4 µm), for unrelated control, patient, and gene-corrected control, respectively (n=30 measurements and N=3 for unrelated control, n=30 measurements and N=1 organoid for the patient, and n=29 measurements and N=2 for isogenic control organoids). E) Rhodopsin signal in the ONL layer of D190 retinal organoids was quantified to be equal to 2.8% (SD = 3.5%), 22.5% (SD = 3.7%), and 1.7% (SD = 1.9%), in unrelated control, patient, and gene-corrected control, respectively (n=5 measurements and N=3 for unrelated control, n=5 measurements and N=1 organoid for patient, and n=5 measurements and N=2 for isogenic control organoids).

Table S1. Primer sequences.

Primer	Primer sequence (5'- 3')
TIDER primer a	CTCCTGCCTAGGCCTCTCAAAGC
TIDER primer b	TCAACCATGCAACACAGTGAAGCT
Alt-R CRISPR-Cas9 crRNA1	GCGACTATATCACAAATTAA
Template gRNA1	ATATGAATAGTAACATTTAGTGTTTTAAAATATTTAATTTTCGTCTAATACAC<br>ATTATGGAAAACAATGCAATTTAAAATACTTACTTTAAGTTTGTGATATAGTC<br>GCTGTACCTCCTTTTGAAGAA
Template 1 TIDER primer d	GTACAGCGACTATATCACAAACTTAAAGTAAGTATTTTAAATTGC
Template 1 TIDER primer c	TTTAAAATACTTACTTTAAGTTTGTGATATAGTCGCTGTACCTCCTTTTGAAG
Alt-R CRISPR-Cas9 crRNA2	GAAATAAAGTTCTTCAAAAGG
Template gRNA2	AAATATTTAATTTTCGTCTAATACACATTATGGAAAACAATGCAATTTAAAAT<br>ACTTACCTTTAATTTGTGATATAGTCGCTGTACTTCCTTTTGCAAAACTTTATT<br>TTCATCATGAGCCTCATATG
Template 2 TIDER primer d	TTTGCAAAAGGAAGTACAGCGACTATATCAC
Template 2 TIDER primer c	GTGATATAGTCGCTGTACTTCCTTTTGCAAAACTTTATTTTCATC
qPCR primer LCA5_ex3_FWD	AGACACCTACCTGAACGAGATG
qPCR primer LCA5_ex4_REV	CTCAGTTCAAGGTTTTTCGATAGC

Table S2. Antibodies.

Antigen	Species	Dilution	Supplier	Catalog number
Opsin Red/Green	Rabbit	1:500	Millipore	AB5405
Lebercilin	Rabbit	1:100	Home-made	n/a
ARL13B	Mouse	1:250	NeuroMab	75-287
Rhodopsin (4D2)	Mouse	1:500	Novus Biologicals	NBP2-59690
Recoverin	Rabbit	1:500	Abcam	ab5585
