# Supplementary material for: CRISPR-Cas9 correction of a nonsense mutation in LCA5 rescues lebercilin expression and localization in human retinal organoids
Source: Mol Ther Methods Clin Dev. 2023 May 17;29:522–31. doi: 10.1016/j.omtm.2023.05.012 (PMC10250556; doi:10.1016/j.omtm.2023.05.012)
Supplement: Document S2. Article plus supplemental information [file mmc2.pdf]

Molecular Therapy
Methods & Clinical Development
Original Article

CRISPR-Cas9 correction of a nonsense mutation in *LCA5* rescues lebercilin expression and localization in human retinal organoids

Tess A.V. Afanasyeva,[1,2,6] Dimitra Athanasiou,[3,6] Pedro R.L. Perdigao,[3] Kae R. Whiting,[1] Lonneke Duijkers,[1] Galuh D.N. Astuti,[1] Jean Bennett,[4] Alejandro Garanto,[1,5] Jacqueline van der Spuy,[3] Ronald Roepman,[1] Michael E. Cheetham,[3] and Rob W.J. Collin[1,2]

[1]Department of Human Genetics, Radboud University Medical Center, 6525 GA Nijmegen, the Netherlands; [2]Donders Institute for Brain, Cognition and Behaviour, Radboud University Medical Center, 6525 GD Nijmegen, the Netherlands; [3]UCL Institute of Ophthalmology, 11-43 Bath Street, London EC1V 9EL, UK; [4]Department of Ophthalmology, Perelman School of Medicine, University of Pennsylvania, Philadelphia PA 19104, USA; [5]Department of Paediatrics, Amalia Children's Hospital, Radboud University Medical Center, 6525 GA Nijmegen, the Netherlands

Mutations in the lebercilin-encoding gene *LCA5* cause one of the most severe forms of Leber congenital amaurosis, an early-onset retinal disease that results in severe visual impairment. Here, we report on the generation of a patient-specific cellular model to study *LCA5*-associated retinal disease. CRISPR-Cas9 technology was used to correct a homozygous nonsense variant in *LCA5* (c.835C>T; p.Q279*) in patient-derived induced pluripotent stem cells (iPSCs). The absence of off-target editing in gene-corrected (isogenic) control iPSCs was demonstrated by whole-genome sequencing. We differentiated the patient, gene-corrected, and unrelated control iPSCs into three-dimensional retina-like cells, so-called retinal organoids. We observed opsin and rhodopsin mislocalization to the outer nuclear layer in patient-derived but not in the gene-corrected or unrelated control organoids. We also confirmed the rescue of lebercilin expression and localization along the ciliary axoneme within the gene-corrected organoids. Here, we show the potential of combining precise single-nucleotide gene editing with the iPSC-derived retinal organoid system for the generation of a cellular model of early-onset retinal disease.

INTRODUCTION

Leber congenital amaurosis (LCA) is a severe and early-onset inherited retinal disease (IRD) that impacts between 1:30,000 to 1:81,000 individuals worldwide, and it is presently largely incurable.[1,2] Lack of access to retinal tissue from patients hinders LCA research.[3] The development of induced pluripotent stem cell (iPSC) technology allows reprogramming of somatic cells into pluripotent stem cell-like state.[4] Isogenic pairs of iPSC lines can be generated owing to advances in genome editing with clustered regularly interspaced short palindromic repeats-CRISPR-associated protein 9 (CRISPR-Cas9) homology-directed repair (HDR) technology. These lines differ only at the gene of interest and can model the phenotype-genotype correlation of inherited diseases in the exact genetic background.[5] In the context of IRD, an iPSC-derived isogenic pair can be differentiated into three-dimensional retina-like structures called retinal organoids (ROs) that, upon a high level of maturation, contain all the neuroretinal cell types, including photoreceptors with inner and outer segment-like structures (OSs) joined by a connecting cilium (CC).[6,7]

One of the most severe forms of LCA is linked to a protein lebercilin, encoded by the gene *Leber Congenital Amaurosis 5 Protein* (*LCA5*) that is localized within the CC of photoreceptor cells.[8,9] Lebercilin interacts with the intraflagellar transport machinery, and in a murine model, its knockout results in the mislocalization of photoreceptor outer segment-specific proteins.[10] A patient with a homozygous nonsense variant (c.835C>T; p.Q279*) in *LCA5* showed symptoms of retinal dystrophy; such as visual loss, nystagmus, and high hyperopia.[9] When iPSCs derived from patient's peripheral blood were differentiated to retinal pigment epithelium cells (RPEs), they had fewer cilia and expressed lower levels of *LCA5* compared with a control.[11] While RPE differentiation yields insights into ciliary phenotype, it cannot recapitulate the cell-specific context of photoreceptors. To our knowledge, no gene-corrected RO model exists for *LCA5*-linked retinal disease.

Here, we corrected a homozygous *LCA5* nonsense variant in patient-derived iPSCs using the CRISPR-Cas9 system and assessed the genome quality in two clones of the generated isogenic control iPSCs with whole-genome sequencing (WGS). Following a 190-day-long differentiation of patient-derived, healthy unrelated, and gene-repaired isogenic control ROs, we were able to confirm the rescue of

Received 11 October 2022; accepted 12 May 2023;
https://doi.org/10.1016/j.omtm.2023.05.012.

[6]These authors contributed equally

Correspondence: Rob W.J. Collin, Department of Human Genetics, Radboud University Medical Center, 6525 GA Nijmegen, the Netherlands.
E-mail: rob.collin@radboudumc.nl

 © 2023 The Author(s).
This is an open access article under the CC BY license (http://creativecommons.org/licenses/by/4.0/).

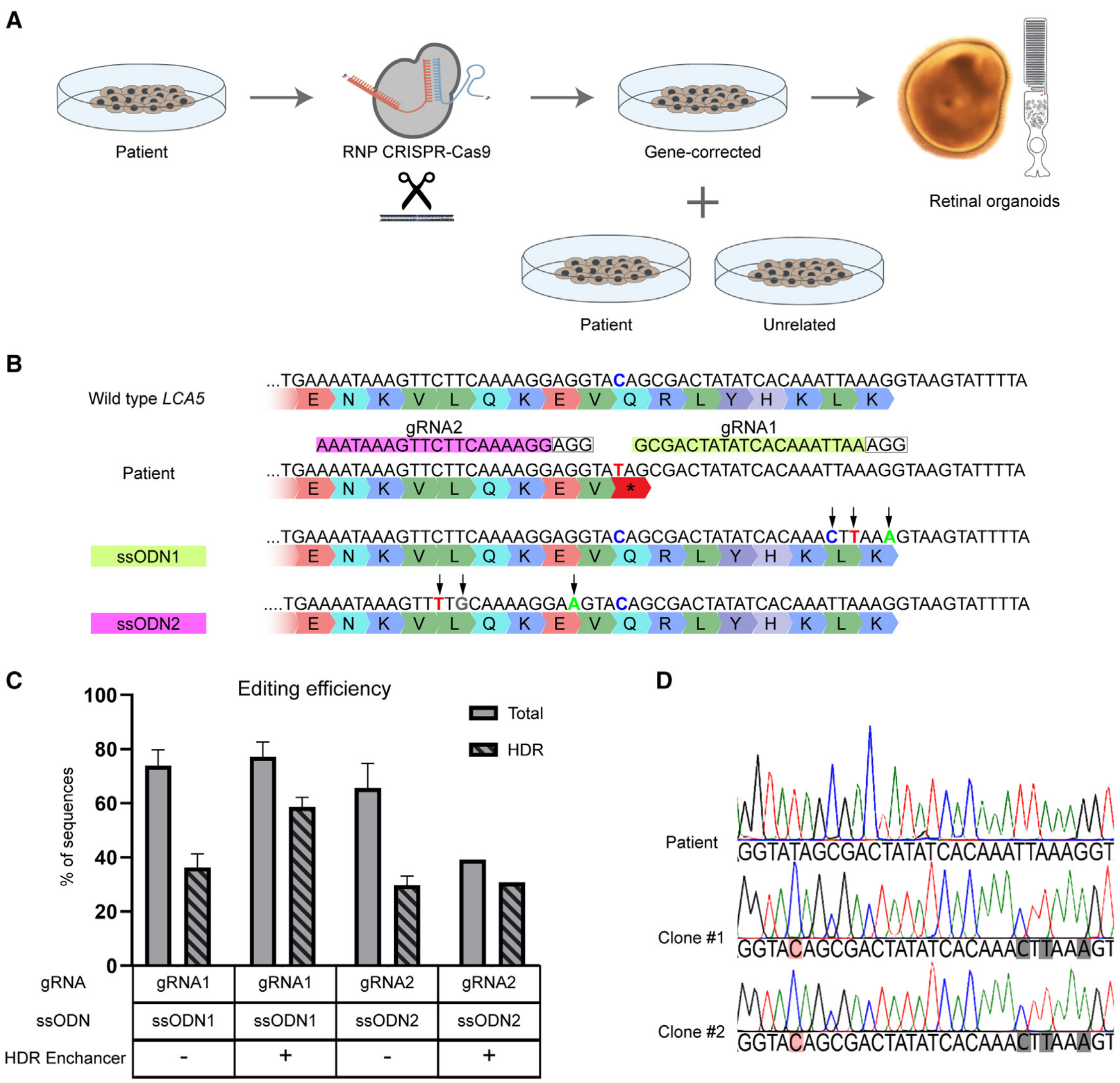

Figure 1. Generation of an isogenic control by CRISPR-Cas9 correction using S.p. Cas9 Nuclease V3 of the homozygous variant *LCA5* c.835C>T in patient-derived iPSCs

(A) Schematic of isogenic pair generation and differentiation into retinal organoids. (B) Two guide RNAs and respective single-stranded oligodeoxynucleotide (ssODNs) (gRNA1 in green, gRNA2 in magenta) targeting the region surrounding the nonsense *LCA5* c.835C>T variant in the patient iPSCs genome to restore the wild-type sequence. Protospacer adjacent motifs (PAM) for each gRNA are in gray. Nucleotide substitutions in ssODN templates are in color, with synonymous nucleotide substitutions signed with arrows. (C) Total (NHEJ+HDR) and *LCA5* c.835C>T correction (HDR) editing efficiencies of gRNA1 and gRNA2 separately or supplemented with 30 μM IDT small-molecule HDR Enhancer (SD is standard deviation, n = 2 experiments, except for gRNA2+HDR Enhancer condition where n = 1). (D) Sanger sequencing of the patient and gene-corrected control clones #1 and #2 that were generated using ssODN1. The c.835C>T substitution is in red, and the three designed PAM-blocking silent nucleotide substitutions are in gray.

lebercilin localization and function in CC of the developing photoreceptors in the gene-edited line.

RESULTS

Generation of an isogenic control for patient iPSC line carrying *LCA5* nonsense variant

We combined iPSC and CRISPR-Cas9 technologies to generate an isogenic control for *LCA5*-associated IRD. We delivered Cas9 ribonucleoprotein (RNP) conjugated with a guide ribonucleic acid (gRNA) targeting the *LCA5* (c.835C>T; p.Q279*) nonsense variant alongside a single-stranded oligodeoxynucleotide (ssODNs) donor template encoding the intended nucleotide correction into patient-derived iPSCs (Figure 1A). We designed two gRNA sequences, followed by the NGG canonical protospacer adjacent motif (PAM) recognized by the Cas9 (Figure 1B). For each gRNA, an ssODN was generated with uneven homologous genomic flanking sequences of 36 and 91 nucleotides

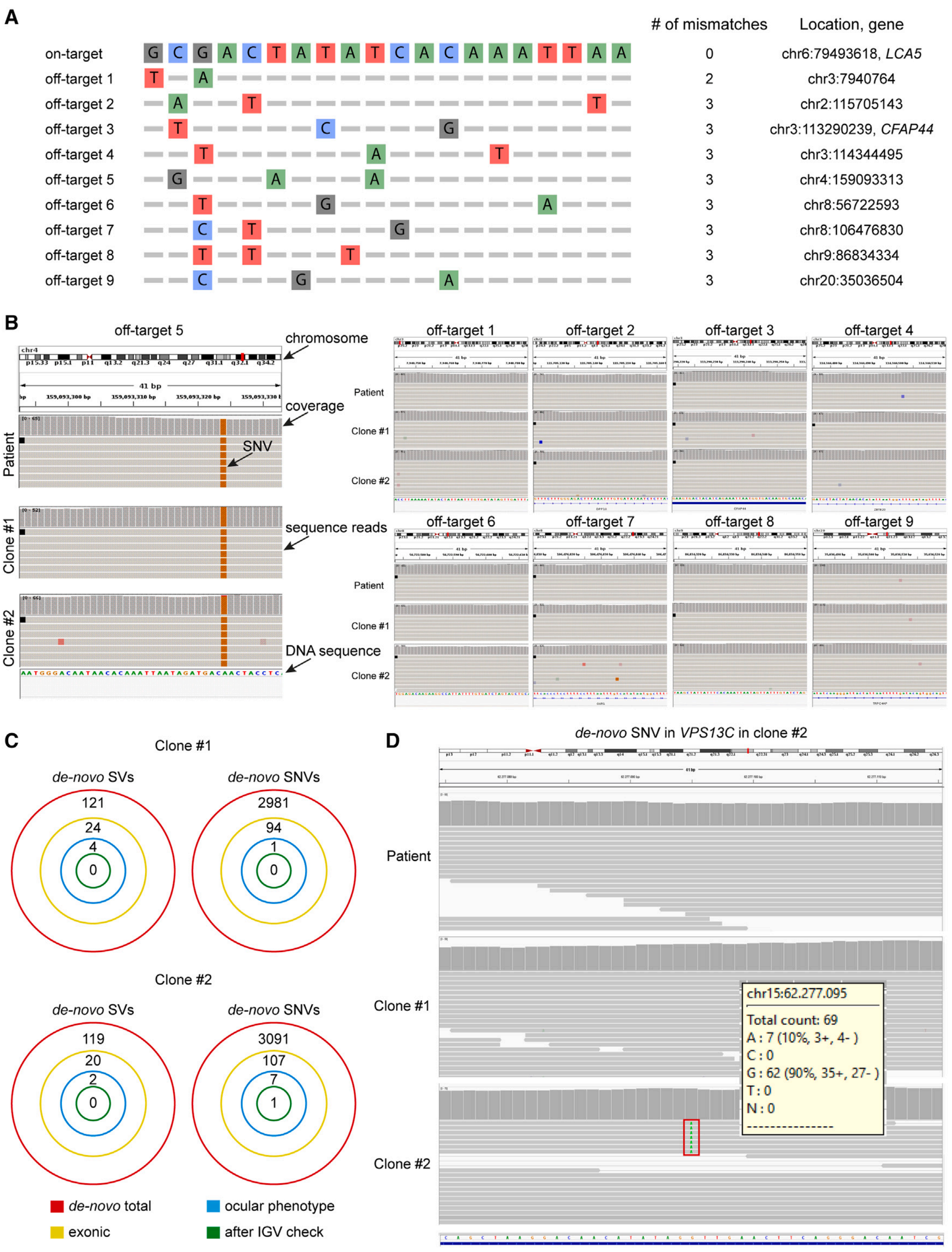

Figure 2. Off-target analysis using whole-genome sequencing

(A) Sequences of nine predicted off-targets of gRNA1 and those containing four or fewer mismatches were selected to be checked with whole-genome sequencing. Protospacer adjacent motifs (PAM), number of mismatches, gene name (if applicable), and position of the off-target, as well as nucleotides differences, are shown. (B) Representative Integrative Genomics Viewer (IGV) 2.4 browser screenshots of four off-target sites with the highest homology. (C) Total, exonic (excluding splice sites variants),

(legend continued on next page)

centered at the predicted CRISPR-Cas9 cleavage site, antisense to the target strand, and containing a desired single-nucleotide substitution together with three silent substitutions in the sequence corresponding to the 3′ end of the gRNA. One substitution changed the canonical PAM sequence (from AGG to A**A**G).[12] We also introduced silent substitutions into the seed regions for both gRNAs. For gRNA1, the seed region sequence directly next to the PAM site was modified in the ssODN (TTAAA into CTTAA). Whereas, for gRNA2, modification of the neighboring sequence of the seed region would have resulted in amino acid change. Therefore, we modified the seed region sequence eight nucleotides upstream of the PAM site (CTTC to TTGC) in the ssODN. In addition to being silent, these changes were predicted not to interfere with pre-mRNA splicing based on *in silico* predictions and subsequent RNA analysis (data not shown). Of the two designed gRNAs, *in silico* analysis showed that gRNA1 had specificity scores (78/100 for gRNA1 vs. 31/100 for gRNA2) and on-target cutting frequency (92/100 for gRNA1 vs. 31/100 for gRNA2), according to the Zhang lab MIT CRISPR design software (https://www.benchling.com/).[13] We evaluated the efficacy using TIDER analysis of the sequenced deoxyribonucleic acid (DNA) in pooled edited iPSCs to determine the frequency of total (non-homologous end-joining [NHEJ] and HDR) and HDR-mediated editing.[14]

As predicted, cells treated with Cas9:gRNA1 RNP and ssODN1 template showed a higher total editing efficiency of 74.0% and 36.3% HDR efficiency (standard deviation [SD] equals 5.9% and 5.0% respectively, n = 2), against 65% and 29.7% HDR in gRNA2 (SD equals 9.6% and 3.4% respectively). Moreover, we improved the HDR efficiency for Cas9:gRNA1/ssODN1 treated cells to 58.7% with the addition of the Alt-R HDR Enhancer molecule (SD equals 3.6%) but not for Cas9:gRNA2/ssODN2 condition (30.87%). We were not able to assess the HDR efficiency of gRNA2 in duplicate due to high cell death of the biological duplicate (Figure 1C). Based on the higher efficiency, we proceeded with Cas9:gRNA1/ssODN1/+Enhancer nucleofection, followed by limiting dilution and clonal selection. Two iPSC clonal lines were isolated where c.835C>T; p.Q279* was corrected to wild-type (T to C) on both *LCA5* alleles (Figure 1D).

Off-target analysis in genome-edited isogenic lines

We quantified gene-editing events in CRISPR-Cas9-corrected clonal lines using next-generation WGS. Firstly, to compare WGS with commonly performed Sanger sequencing off-target analysis, we predicted *in silico* the off-target editing sites with the highest homology to the gRNA1 target sequence, restricted to four or fewer mismatches.[13] Of nine predicted sites, one was located in a gene-coding region in *CFAP44* (Figure 2A). We assessed DNA sequences at the genomic coordinates of the predicted sites in the *LCA5* c.835C>T iPSC line, which from here on will be referred to as patient or "parental," and two gene-corrected iPSC lines (clone #1 and clone #2) and detected no off-targets (Figure 2B).

Next, we searched for genomic alterations that could have been introduced by CRISPR-Cas9 editing, i.e., genome sequences that were different between edited iPSCs lines and the parental line. Alterations could include structural variants (SVs), such as insertions deletions, or inversions, or they could include single-nucleotide variants (SNVs), such as single-nucleotide insertions, deletions, and substitutions. We employed an automated bioinformatic detection pipeline to detect the genomic changes. These predicted changes were then assessed manually using visualization with the Integrative Genomics Viewer (IGV) software. The automatic analysis detected 121 and 119 SVs in clones #1 and #2, respectively. Upon manual examination, all SVs were shown to be false positives, as they were also present in the parental line prior to editing and were miscalled by the pipeline as being different. Subsequently, the genomes of clones #1 and #2 were checked for SNVs. In total, in clone #1, 94 *de novo* SNVs were predicted, whereas in clone #2, 107 were suggested to be present in the edited lines, but not in the parental line. Of these, the SNVs, one SNV in clone #1 and seven SNVs in clone #2 were detected in ocular phenotype-associated genes (Figure 2C). However, manual examination of all the SNVs in IGV did not validate the vast majority of these calls from the bioinformatic pipeline; only one *de novo* substitution (G to A) in exon 17 was confirmed in the ocular phenotype-associated gene *VPS13* on chromosome 15 in clone #2. This was identified in 10% of the sequences (Figure 2D), suggesting this might be low-quality call or a mixed clone. No SNVs were identified in clone #1, with the correction of the *LCA5* c.835C>T allele and gRNA1 substitutions as the only identified changes from the parental line. In conclusion, WGS analysis showed an SNV in clone# 2 and no off-target editing in clone #1, which was used for the remainder of this study. In addition, all iPSC lines presented a normal karyotype and expression of pluripotency markers (Figure S1).

Rescue of lebercilin expression and ciliary localization in *LCA5* c.835C>T corrected isogenic iPSCs

We differentiated the patient, the isogenic gene-corrected, and unrelated control iPSCs for 120, 150, and 190 days (D120, D150, and D190) into ROs (Figure 3A). At D190, we observed no visible deviations in lamination or photoreceptor OSs between patient, gene-corrected, and control lines by light microscopy (Figure S2A). Organoids of all lines contained a photoreceptor-marker positive outer layer (Figure S2B), where the average thickness was 25.8 μm (SD = 6.4 μm), 32.4 μm (SD = 7.9 μm), and 31.2 μm (SD = 8.4 μm), for unrelated control, patient, and gene-corrected control, respectively (Figure S2D). To confirm the lebercilin expression and ciliary localization,

ocular phenotype-linked, and post-manual evaluation *de novo* structural variants (SVs) and single-nucleotide variants (SNVs) in isogenic control clones #1 and #2 are represented schematically. (D) IGV 2.4 browser screenshots of *de novo* substitution (G to A) in exon 17 of the VPS13C gene on chromosome 15 in clone 2. (B) and (D) show the 41-bp sequence around the site of interest, as well as the chromosome, sequence, and name of the gene in which it is located, sequence reads, and coverage of the *LCA5* c.835C>T and isogenic control clone #1 and #2.

we performed immunohistochemistry (IHC) with lebercilin and ciliary marker-specific antibodies. In the unrelated control line, lebercilin was localized in the proximity of ciliary markers. We did not detect any protein expression above the background in the patient-derived ROs at any time point. At the same time point, protein expression and ciliary localization of lebercilin were evident in isogenic gene-corrected ROs (Figure 3B). Furthermore, in the isogenic control, expression levels of *LCA5* mRNA were restored to those of the healthy control at D120 and D150 (Figure S2C). At D120, in non-isogenic control, the gene expression of *LCA5* was 22.9-fold higher than in the patient-derived organoids (SD = 4.6). In the isogenic control, the gene expression was rescued to the level of non-isogenic (24.2 fold change [FC], SD = 2.2). At D150, it decreased in both non-isogenic and isogenic (13.6 FC, SD = 1.0 and 15.1 FC, SD = 0.3). At D190, it decreased for isogenic (4.9 FC, SD = 0.2) and less dramatically for non-isogenic control (10.1 FC, SD = 1.1).

In the mature ROs, photoreceptors possess the OSs harboring rhodopsin and opsins.[7] At D190, IHC showed rhodopsin-positive OSs in all lines; however, in the patient-derived organoids, rhodopsin was also detected in the outer nuclear layer (ONL) (Figure S2B). We quantified rhodopsin intensity in the ONL of ROs of all three lines, where the average area of rhodopsin staining against the area of recoverin staining in ONL was equal to 2.8% (SD = 3.5%), 22.5% (SD = 3.7%), and 1.7% (SD = 1.9%), in unrelated control, patient, and gene-corrected control, respectively (Figure S2E). Likewise, at D190, long-wave sensitive cone opsin was present in the OSs of all three lines (Figure 3C). Due to the lower frequency of cone-positive cells in the ROs of all lines, we could not quantify the retention of cone opsin in ONL.

DISCUSSION

Patient-derived iPSC ROs offer opportunities for a deeper understanding of the pathophysiological mechanisms involved in IRDs. In this study, we corrected a nonsense variant in the *LCA5* gene in the genome of patient-derived iPSCs. We observed ciliary localization of lebercilin in ROs differentiated from the unrelated control line and the isogenic gene-corrected line but not the patient line. Moreover, opsin and rhodopsin mislocalization into the ONL was rescued in the gene-corrected organoids.

LCA5-associated LCA is recessively inherited, which makes it an excellent candidate for genome editing. We designed two gRNAs in a genomic region adjacent to a causative nonsense variant c.835C>T; p.Q279* in exon 5 of *LCA5*. Of these, gRNA1 had a higher predicted specificity score but also a disadvantage of a longer cut-to-variant distance, with a CRISPR-Cas9 cleavage site at 19 nucleotides downstream of the variant, compared with the nine nucleotides of the gRNA2. A shorter cut-to-variant distance has been previously shown to improve the probability of homozygous corrections.[15] To estimate the efficiency of editing, we analyzed pooled corrected iPSC clones with the TIDER tool, which showed a high editing efficiency for gRNA1. We did not observe a decrease in cell viability post editing, as we used the RNP delivery that improves the efficiency and precision of HDR editing while decreasing cytotoxicity.[16] The addition of Alt-R Enhancer increased the HDR editing efficiency, but it also increased cytotoxicity for the gRNA2.

Off-target editing can introduce a confounding phenotype that complicates the interpretation of the phenotype of a cellular model and is a concern in the clinical application of gene editing. WGS allows the detection of off-targets throughout the complete genome.[17] In two gene-edited iPSCs clones, we did not detect insertions, deletions, or other SVs. In several cases, we detected false-positive SNVs and SVs already present in the parental line using a bioinformatic pipeline. The genomic variability of iPSCs could result from reprogramming of the somatic cells or random genetic drift, common for cells maintained in culture.[18] One *de novo* SNV was confirmed in clone #2, in gene *VPS13C*, which is linked to Parkinson's disease and an eye phenotype in mice.[19] A small number of sequences included the substitution indicating a potential heterozygous change, the presence of a mixed clone, or low-quality call. This potential off-target could be facilitated by nearby regions with high homology for gRNA1. Two nearby genomic regions, 145 nucleotides downstream and 186 nucleotides upstream of the SNV, showed seven-nucleotide-long homologies with the gRNA1 target sequence, which suggest toward a higher chance of NHEJ editing. NHEJ, however, tends to cause insertions, deletions, and inversions that are larger than one nucleotide. Instead, a single-nucleotide substitution could be a spontaneous mutation in a clone or a part of a clone. This finding underlines the importance of assessing the quality of the complete genome.

ROs recapitulate early stages of development and are suitable to model early-onset LCA. We differentiated *LCA5* c.835C>T, isogenic gene-corrected, and unrelated control iPSCs into ROs.[20–22] It is worth highlighting that the unrelated control stem cells were male with X and Y chromosomes, while the isogenic pair were female, with two X chromosomes. A limited number of control iPSC lines have been generated from peripheral blood cells using Sendai virus reprogramming, which makes it challenging to match the lines. It further underlines the importance of the generation of isogenic control lines that carry the same genotype as the patient. In this case, however, no previous differences in expression and localization of lebercilin were reported between different sexes. We investigated the expression and localization of lebercilin in the photoreceptor CC. Gene expression analysis showed low background levels of *LCA5* expression in patient-derived RO, which was rescued in the isogenic control RO to the level of a healthy control at D120 and D150. At the D190, *LCA5* expression decreased for both healthy and isogenic controls, with the latter showing several folds lower expression. The general decrease of *LCA5* transcript in mature RO has been previously reported to occur around day 168.[23] Lebercilin was expressed in the cilia at D120 when photoreceptor maturation begins and in the CC of photoreceptors at D150 and D190 in gene-edited ROs, as well as in those derived from the unrelated control. At all observed time points, the *LCA5* c.835C>T ROs showed no

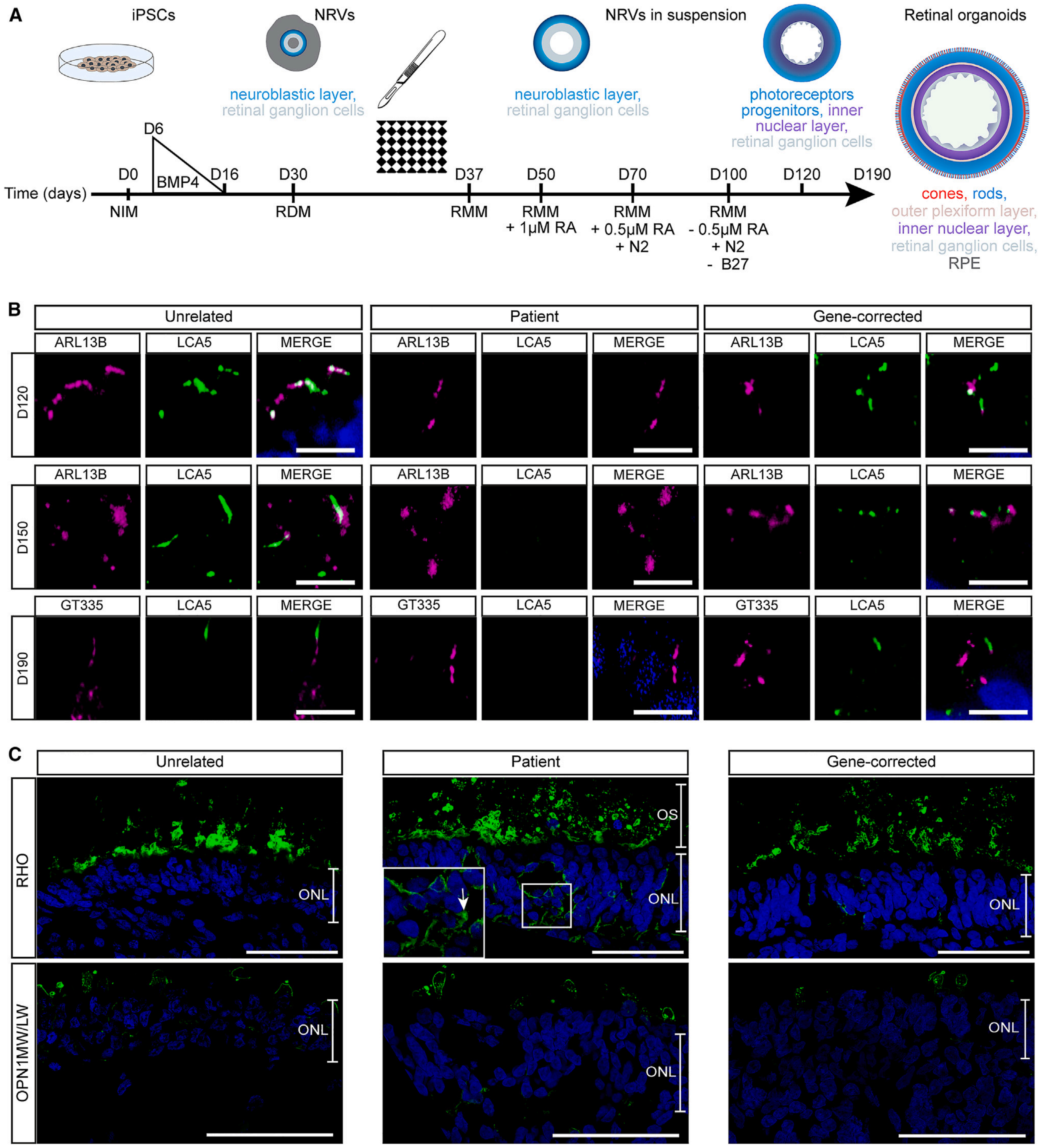

Figure 3. Expression and localization of lebercilin in *LCA5* c.835C>T, gene-corrected, and unrelated control human ROs

(A) Adapted schematic representation of retinal organoids (ROs) differentiation protocol used for this study[15–17]. BMP4 is bone morphogenetic protein 4, NRV is a neural retinal vesicle, NIM is neural induction media, RDM is retinal differentiation media, RMM is retinal maturation media, RA is retinoic acid, RPE is retinal pigment epithelium, N2 designates N2 supplement, and B27 designates B27 supplement. (B) Representative images of lebercilin localization in days D120, D150, and D190 ROs. Lebercilin is in green, ciliary markers ARL13B and GT335 in magenta, and nuclear stain DAPI in blue. (C) Immunohistochemical analysis demonstrating rod and cone marker localization in D190 ROs. Top panel: rod marker RHO is in green and nuclear stain DAPI in blue. Bottom panel: cone marker OPN1MW/OPN1LW is in green and nuclear stain DAPI in blue. White arrows point at the rhodopsin signal in ONL. Scale bars: 5 μm (B); 50 μm (F).

lebercilin expression, consistent with observations in RPE cells.[11] For D190 ROs, we used the ciliary marker GT335, due to ARL13B localization into the outer segments.[24] In photoreceptor cells of D190 ROs, the absence of lebercilin caused rhodopsin and opsin retention in the ONL in line with the phenotype of the *Lca5*$^{-/-}$ mouse model.[10] We observed a lower level of rhodopsin and opsin intensity in the ONL of the gene-edited ROs, which points toward the potential rescue of lebercilin function. Interestingly, ONL nuclei were observed trapped in the OS layer of the patient's ROs, which can signify a cell death phenotype[25]; however, this could also be a cryosectioning artifact. A follow-up study is required to elucidate the full effect of gene editing on the lebercilin function.

This study demonstrates the value of generating gene-corrected isogenic controls to model IRDs by reporting the correction of a causative variant in patient-derived iPSCs and the rescue of *LCA5* gene expression, protein localization, and function. The described cellular model provides a foundation for understanding the molecular mechanisms underlying *LCA5*-associated IRD and evaluating potential therapeutic approaches.

MATERIALS AND METHODS

Maintenance of iPSCs lines

Patient-derived iPSCs with a homozygous nonsense variant (c.835C>T; p.Q279*) in exon 5 were a kind gift from Prof. Jean Bennett (University of Philadelphia, USA) and were characterized previously.[11] The control iPSC line was a kind gift of Prof. Oliver Brüstle. Both iPSC lines were derived from peripheral blood and transduced with Sendai viral vectors expressing *Oct3/4, SOX2, KLF4*, and *cMYC* (MOIs according to manufacturer's instructions, Thermo Fisher). iPSCs were defrosted on laminin-coated rh-Laminin-521 (Gibco, USA) six-well plates (CC7682-7506, CytoOne) in StemFlex-supplemented medium (Gibco, USA) and split using enzymatic dissociation with Cell Dissociation Buffer, enzyme-free, PBS (Gibco, USA). After one passage, iPSCs were maintained and expanded on GelTrex-coat in Essential 8 Flex supplemented basal culture medium (all Gibco, USA).

Generation of isogenic control lines

Genomic DNA was isolated with the Wizard SV Genomic DNA Purification kit (A2361, Promega). Presence of the *LCA5* c.835C>T variant in the iPSCs was validated by amplification with primers 5′-CTCCTGCCTAGGCCTCTCAAAGC-3′ (forward) and 5′-TCAA CCATGCAACACAGTGAAGCT-3′ (reverse). PCR reactions were carried out with 100 ng genomic DNA in GoTaq Green Master Mix (M7122, Promega) according to the manufacturer's instructions. PCR thermocycling scheme was as follows: 5 min 95°C (1×), followed by 20 s at 95°C, 30 s at 62°C and 45 s at 72°C (35×) and 5 min at 72°C (1×). PCR products were purified using MultiScreen PCRμ96 Filter Plate (Merck, USA), and the mutation was confirmed by Sanger sequencing. To target exon 5 of *LCA5* gene (c.835C>T; p.Q279*), gRNAs and ssODNs were designed, and potential off-target sites were predicted using the Benchling tool (www.benchling.com [13,26]). Sequences of gRNAs and ssODNs are available in Table S1. iPSCs were supplemented with RHO/ROCK pathway inhibitor (20 μM) in StemFlex 2 h before nucleofection. CRISPR-Cas9 tracrRNA (IDT, USA) and Alt-R CRISPR-Cas9 crRNA were resuspended in Nuclease-Free Duplex Buffer IDT (IDT, USA) and annealed to produce 50 μM concentration gRNA. 150 pmol of gRNA and 125 pmol of Alt-R S.p. Cas9 Nuclease V3 (IDT, USA) per one nucleofection reaction were incubated at room temperature (RT) for 20 min to form an RNP complex. Ultramer DNA Oligo ssODN donor strand was reconstituted in RNAase-free water to a concentration of 100 μM. RNP complex, ssODN donor, and Alt-R Cas9 Electroporation Enhancer (IDT, USA) were combined with the 0.2 x 10^6 cells per nucleofection reaction in Primary P3 Solution of P3 Primary Cell 4D-Nucleofector X Kit S and transfected according to the manufacturer's instructions (Lonza, Switzerland). Optionally, 30 μM HDR Enhancer (IDT, USA) was added to the culture media. 4 days after transfection, single-cell-derived clones were obtained by limiting dilution. Pooled sequences were analyzed with TIDER web tool.[14] Rstudio version 4.1.0 and GraphPad Prism 6 software were used for statistical analysis. Furthermore, a portion of single-cell derived colonies was manually detached, and DNA was isolated with QuickExtract DNA Extraction Solution (Lucigen, USA). Sanger sequencing was used to select corrected clones that had been obtained by limiting dilution, and the indicated clones were subjected to WGS.

Pluripotency analysis

Pluripotency analysis was performed by Radboudumc Stem Cell Technology Center according to standard procedures. Karyotyping was performed following standard protocols as applied in cytogenetic laboratories. Briefly, cultured IPS cells were collected in 1.5-mL tubes. Metaphase arrest was then induced using colcimid followed by a hypotonic treatment followed by fixation with an appropriate mixture of ethanol and acetic acid. Metaphases were spread out on glass slides under controlled temperature and humidity using a Hanabi PV automated system (ADS Biotec). Subsequently, chromosomes were GTG stained before analyzing with, respectively, trypsin and heating-based denaturation. Image capturing was automated using a karyotype imaging system (Leica Cytovision System, Amsterdam, the Netherlands). Chromosomal abnormalities were described according to the International System for Human Cytogenetic Nomenclature (ISCN, 2020).

Whole-genome sequencing

Genomic DNA of the patients' and two CRISPR-corrected isogenic controls were prepared using the Qiagen DNeasy Blood & Tissue Kit (Qiagen, Germany), and genome sequencing was performed on the BGIseq-500 sequencer at an average depth of 30-fold per sample. Sequenced reads were aligned to the Human Reference Genome (GRCh37.p5/hg19) using BWA-Mem[27] SNVs, and small indels were identified and quality-filtered using xAtlas,[28] and SVs were detected using CANVAS.[29] The predicted SVs, SNVs, and off-target areas were manually inspected using Integrative Genomics Viewer 2.4 software.

Retinal organoid differentiation

The patient-derived iPSC, gene-repaired isogenic, and non-isogenic control lines were differentiated as previously described.[20–22] Briefly, Essential 6 Medium (Gibco, USA) was added to the iPSCs at day 0 (D0) and refreshed after 24 h. On D2, medium was changed to neural induction media (NIM), consisting of Advanced DMEM and Ham's F-12 Nutrient Mix (1:1) supplemented with 1% N2 supplement and 1% GlutaMAX (all Gibco, USA). At D6, 1.5 nM bone morphogenetic protein 4 (BMP4) per well was added and half media changed until D16, after which full media was changed three times per week. The appeared neural retinal vesicles (NRVs) were dissected with a scalpel and transferred using a 1-mL pipette with a cut tip into one well of a 96-well low attachment plate (Corning, USA). The remaining structures were detached using the checkerboard scraping method.[23] Once all the NRVs were transferred, the medium was changed to retinal differentiation media (RDM), which consisted of DMEM and Ham's F-12 Nutrient Mix (3:1) supplemented with 2% B27 (without Vitamin A) and 1% NEAA (all Gibco, USA). 7 days after (D37), the medium was changed to retinal maturation media (RMM), which consisted of DMEM and Ham's F-12 Nutrient Mix (3:1) supplemented with 10% FBS, 2% B27 (without Vitamin A), 100 μM taurine (Sigma-Aldrich, USA), and 2 mM Glutamax (all Gibco, USA, with exceptions). At D50, the RMM was supplemented with 1 μM 9-*cis*-retinal or RA (Sigma-Aldrich, USA). At D70, RMM was further supplemented with 1% N2, and 9-*cis*-retinal concentration was lowered to 0.5 μM. At D100, 9-*cis*-retinal and B27 (without vitamin A) supplements were removed. All media were supplemented with 1% Antibiotic Antimycotic (Gibco, USA).

RNA extraction and quantitative real-time PCR

ROs (n = 3 ROs per measurement, in duplicate) were subjected to RNA extraction using RNeasy MicroKit (Qiagen, Netherlands), and cDNA synthesis was performed using Invitrogen SuperScript VILO cDNA Synthesis Kit (Invitrogen, USA) for reverse transcription. Primers were designed to cross exon boundaries (Table S1). For quantitative real-time PCR (qPCR) analyses, the GoTaq qPCR master mix (Promega, USA) was used according to manufacturer's instructions, and the analysis was carried out on an Applied Biosystems QuantStudio 3 Real-Time PCR System using the SYBR Green method. Ct values were evaluated in QuantStudio Design and Analysis Software 1.4.3. Data from the qPCR were normalized to expression of *GUSB* gene and *LCA5* expression of the patient line. Error bars represent SD. Statistical tests were performed using GraphPad Prism 9.0.0 (121).

Immunohistochemistry

One differentiation was performed and two ROs were stained per condition. The organoids were positioned in the cryomold so that the OSs were parallel to the knife of the cryosectioning machine; this is done during the cryomold preparation by orienting the RPE patch toward the researcher, as this allows us to obtain longer outer segment-inhabited patches that are assessed by eye under an EVOS microscope. The position of outer segments is further confirmed during the cryosectioning on one of the slides using bright-field microscopy. Immunohistochemistry was performed as previously described.[30] The staining for *LCA5* and the ciliary marker are performed simultaneously on the same organoid/slide. Briefly, sections were first washed once in PBS and blocked with 10% donkey serum and 0.05% Triton X-100 in PBS for 1 h at RT. Primary antibodies were incubated in the blocking solution 50% diluted in PBS for 1 h at RT. Sections were then washed in PBS and incubated with Alexa Fluor secondary antibodies in the diluted blocking solution for 45 min at RT. Samples were washed in PBS and mounted using Vectashield Antifade Mounting Media (Vector Laboratories, USA). The list of antibodies used in this study is presented in Table 2. All images were obtained using a Carl Zeiss LSM900 laser-scanning confocal microscope. Images were exported from Zen 3.1 software and combined using Adobe Illustrator 2022. For quantification of ONL thickness, we quantified n = 30 measurements and N = 3 for unrelated control, n = 30 measurements and N = 1 organoid for the patient, and n = 29 measurements and N = 2 for isogenic control organoids. For quantification of the percentage of rhodopsin-positive cells in ONL, we divided the area of rhodopsin-positive cells to the area of recoverin-positive cells in ONL (n = 5 measurements and N = 3 for unrelated control, n = 5 measurements and N = 1 organoid for patient, and n = 5 measurements and N = 2 for isogenic control organoids). The analysis was performed in Fiji 1.47 and GraphPad Prism 9.

DATA AVAILABILITY

The data that support the findings of this study are available from the corresponding author, R.W.J.C, upon reasonable request.

SUPPLEMENTAL INFORMATION

Supplemental information can be found online at https://doi.org/10.1016/j.omtm.2023.05.012.

ACKNOWLEDGMENTS

This work was supported by ZonMW TOP grant (grant number 9121 6051) to R.R. and R.W.J.C.; the Foundation Fighting Blindness USA (grant numbers PPA-0517-0717-RAD to R.W.J.C. and A.G. and FFB-PPA-1719-RAD to R.R., M.E.C., and D.A.); Landelijke Stichting voor Blinden en Slechtzienden to R.R. and R.W.J.C.; Stichting Retina Nederland Fonds to R.R. and R.W.J.C.; Stichting Beheer Het Schild to R.R. and R.W.J.C.; Stichting Blinden-Penning to R.R. and R.W.J.C.; Stichting Steunfonds Uitzicht (Uitzicht grant numbers 2016/2017-22, 2019-17) to R.R. and R.W.J.C.; Gelderse Blindenstichting to R.R. and R.W.J.C.; Rotterdamse Stichting Blindenbelangen to R.R. and R.W.J.C.; Stichting tot Verbetering van het Lot der Blinden to R.R. and R.W.J.C.; Stichting voor gehandicapte kinderen Dowilvo to R.R. and R.W.J.C.; and the Proefdiervrij project support to T.A.V.A. The Wellcome Trust (205041) and Fight for Sight to M.E.C. Moorfields Eye Charity and MRC to M.E.C. and J.v.d.S. The funding organizations had no role in the design or conduct of this review. They provided unrestricted grants. The unrelated control iPSC cell line was a kind gift from Dr. Brüstle.

AUTHOR CONTRIBUTIONS

T.A.V.A.: conceptualization, methodology, formal analysis, investigation, visualization, and writing – original draft; D.A.: conceptualization, methodology, investigation, resources, and review & editing; P.R.L.P.: conceptualization, methodology, investigation, and review & editing; K.R.W.: methodology and investigation; L.D.: methodology and investigation; G.D.N.A.: software and formal analysis; J.B.: resources; A.G.: conceptualization, resources, and review & editing; J.v.d.S.: conceptualization, funding acquisition, and review & editing; R.R.: conceptualization, resources, funding acquisition, and review & editing; M.E.C.: conceptualization, resources, funding acquisition, validation, and review & editing; R.W.J.C.: conceptualization, resources, validation, funding acquisition, and writing – review & editing.

DECLARATION OF INTERESTS

The authors declare that they have no conflict of interest related to the content of this manuscript.

REFERENCES

1. Koenekoop, R.K. (2008). Successful RPE65 gene replacement and improved visual function in humans. Ophthalmic Genet. *29*, 89–91. https://doi.org/10.1080/13816810802216480.
2. Stone, E.M. (2007). Leber congenital amaurosis - a model for efficient genetic testing of heterogeneous disorders: LXIV Edward Jackson Memorial Lecture. Am. J. Ophthalmol. *144*, 791–811. https://doi.org/10.1016/j.ajo.2007.08.022.
3. Hufnagel, R.B., Ahmed, Z.M., Corrêa, Z.M., and Sisk, R.A. (2012). Gene therapy for Leber congenital amaurosis: advances and future directions. Graefes Arch. Clin. Exp. Ophthalmol. *250*, 1117–1128. https://doi.org/10.1007/s00417-012-2028-2.
4. Takahashi, K., Tanabe, K., Ohnuki, M., Narita, M., Ichisaka, T., Tomoda, K., and Yamanaka, S. (2007). Induction of pluripotent stem cells from adult human fibroblasts by defined factors. Cell *131*, 861–872. https://doi.org/10.1016/j.cell.2007.11.019.
5. Bassett, A.R. (2017). Editing the genome of hiPSC with CRISPR/Cas9: disease models. Mamm. Genome *28*, 348–364. https://doi.org/10.1007/s00335-017-9684-9.
6. Afanasyeva, T.A.V., Corral-Serrano, J.C., Garanto, A., Roepman, R., Cheetham, M.E., and Collin, R.W.J. (2021). A look into retinal organoids: methods, analytical techniques, and applications. Cell. Mol. Life Sci. *78*, 6505–6532. https://doi.org/10.1007/s00018-021-03917-4.
7. Zhong, X., Gutierrez, C., Xue, T., Hampton, C., Vergara, M.N., Cao, L.H., Peters, A., Park, T.S., Zambidis, E.T., Meyer, J.S., et al. (2014). Generation of three-dimensional retinal tissue with functional photoreceptors from human iPSCs. Nat. Commun. *5*, 4047. https://doi.org/10.1038/ncomms5047.
8. Dharmaraj, S., Li, Y., Robitaille, J.M., Silva, E., Zhu, D., Mitchell, T.N., Maltby, L.P., Baffoe-Bonnie, A.B., and Maumenee, I.H. (2000). A novel locus for Leber congenital amaurosis maps to chromosome 6q. Am. J. Hum. Genet. *66*, 319–326. https://doi.org/10.1086/302719.
9. den Hollander, A.I., Koenekoop, R.K., Mohamed, M.D., Arts, H.H., Boldt, K., Towns, K.V., Sedmak, T., Beer, M., Nagel-Wolfrum, K., McKibbin, M., et al. (2007). Mutations in LCA5, encoding the ciliary protein lebercilin, cause Leber congenital amaurosis. Nat. Genet. *39*, 889–895. https://doi.org/10.1038/ng2066.
10. Boldt, K., Mans, D.A., Won, J., van Reeuwijk, J., Vogt, A., Kinkl, N., Letteboer, S.J.F., Hicks, W.L., Hurd, R.E., Naggert, J.K., et al. (2011). Disruption of intraflagellar protein transport in photoreceptor cilia causes Leber congenital amaurosis in humans and mice. J. Clin. Invest. *121*, 2169–2180. https://doi.org/10.1172/jci45627.
11. Song, J.Y., Aravand, P., Nikonov, S., Leo, L., Lyubarsky, A., Bennicelli, J.L., Pan, J., Wei, Z., Shpylchak, I., Herrera, P., et al. (2018). Amelioration of neurosensory structure and function in animal and cellular models of a congenital blindness. Mol. Ther. *26*, 1581–1593. https://doi.org/10.1016/j.ymthe.2018.03.015.
12. Richardson, C.D., Ray, G.J., DeWitt, M.A., Curie, G.L., and Corn, J.E. (2016). Enhancing homology-directed genome editing by catalytically active and inactive CRISPR-Cas9 using asymmetric donor DNA. Nat. Biotechnol. *34*, 339–344. https://doi.org/10.1038/nbt.3481.
13. Hsu, P.D., Scott, D.A., Weinstein, J.A., Ran, F.A., Konermann, S., Agarwala, V., Li, Y., Fine, E.J., Wu, X., Shalem, O., et al. (2013). DNA targeting specificity of RNA-guided Cas9 nucleases. Nat. Biotechnol. *31*, 827–832. https://doi.org/10.1038/nbt.2647.
14. Brinkman, E.K., and van Steensel, B. (2019). Rapid quantitative evaluation of CRISPR genome editing by TIDE and TIDER. In CRISPR Gene Editing: Methods and Protocols, Y. Luo, ed. (Springer New York), pp. 29–44. https://doi.org/10.1007/978-1-4939-9170-9_3.
15. Paquet, D., Kwart, D., Chen, A., Sproul, A., Jacob, S., Teo, S., Olsen, K.M., Gregg, A., Noggle, S., and Tessier-Lavigne, M. (2016). Efficient introduction of specific homozygous and heterozygous mutations using CRISPR/Cas9. Nature *533*, 125–129. https://doi.org/10.1038/nature17664.
16. Kim, S., Kim, D., Cho, S.W., Kim, J., and Kim, J.S. (2014). Highly efficient RNA-guided genome editing in human cells via delivery of purified Cas9 ribonucleoproteins. Genome Res. *24*, 1012–1019. https://doi.org/10.1101/gr.171322.113.
17. Gilissen, C., Hehir-Kwa, J.Y., Thung, D.T., van de Vorst, M., van Bon, B.W.M., Willemsen, M.H., Kwint, M., Janssen, I.M., Hoischen, A., Schenck, A., et al. (2014). Genome sequencing identifies major causes of severe intellectual disability. Nature *511*, 344–347. https://doi.org/10.1038/nature13394.
18. Popp, B., Krumbiegel, M., Grosch, J., Sommer, A., Uebe, S., Kohl, Z., Plötz, S., Farrell, M., Trautmann, U., Kraus, C., et al. (2018). Need for high-resolution genetic analysis in iPSC: results and lessons from the ForIPS consortium. Sci. Rep. *8*, 17201. https://doi.org/10.1038/s41598-018-35506-0.
19. Lesage, S., Drouet, V., Majounie, E., Deramecourt, V., Jacoupy, M., Nicolas, A., Cormier-Dequaire, F., Hassoun, S.M., Pujol, C., Ciura, S., et al.; French Parkinson's Disease Genetics Study PDG; International Parkinson's Disease Genomics Consortium IPDGC (2016). Loss of VPS13C function in autosomal-recessive parkinsonism causes mitochondrial dysfunction and increases PINK1/parkin-dependent mitophagy. Am. J. Hum. Genet. *98*, 500–513. https://doi.org/10.1016/j.ajhg.2016.01.014.
20. Reichman, S., Terray, A., Slembrouck, A., Nanteau, C., Orieux, G., Habeler, W., Nandrot, E.F., Sahel, J.A., Monville, C., and Goureau, O. (2014). From confluent human iPS cells to self-forming neural retina and retinal pigmented epithelium. Proc. Natl. Acad. Sci. USA *111*, 8518–8523. https://doi.org/10.1073/pnas.1324212111.
21. Gonzalez-Cordero, A., Kruczek, K., Naeem, A., Fernando, M., Kloc, M., Ribeiro, J., Goh, D., Duran, Y., Blackford, S.J.I., Abelleira-Hervas, L., et al. (2017). Recapitulation of human retinal development from human pluripotent stem cells generates transplantable populations of cone photoreceptors. Stem Cell Rep. *9*, 820–837. https://doi.org/10.1016/j.stemcr.2017.07.022.
22. Capowski, E.E., Samimi, K., Mayerl, S.J., Phillips, M.J., Pinilla, I., Howden, S.E., Saha, J., Jansen, A.D., Edwards, K.L., Jager, L.D., et al. (2019). Reproducibility and staging of 3D human retinal organoids across multiple pluripotent stem cell lines. Development *146*, dev171686. https://doi.org/10.1242/dev.171686.
23. Cowan, C.S., Renner, M., De Gennaro, M., Gross-Scherf, B., Goldblum, D., Hou, Y., Munz, M., Rodrigues, T.M., Krol, J., Szikra, T., et al. (2020). Cell types of the human retina and its organoids at single-cell resolution. Cell *182*, 1623–1640.e34. https://doi.org/10.1016/j.cell.2020.08.013.
24. Hanke-Gogokhia, C., Wu, Z., Sharif, A., Yazigi, H., Frederick, J.M., and Baehr, W. (2017). The guanine nucleotide exchange factor Arf-like protein 13b is essential for assembly of the mouse photoreceptor transition zone and outer segment. J. Biol. Chem. *292*, 21442–21456. https://doi.org/10.1074/jbc.RA117.000141.
25. Gasparini, S.J., Tessmer, K., Reh, M., Wieneke, S., Carido, M., Völkner, M., Borsch, O., Swiersy, A., Zuzic, M., Goureau, O., et al. (2022). Transplanted human cones incorporate and function in a murine cone degeneration model. J. Clin. Invest. *132*, e154619. https://doi.org/10.1172/JCI154619.
26. Doench, J.G., Fusi, N., Sullender, M., Hegde, M., Vaimberg, E.W., Donovan, K.F., Smith, I., Tothova, Z., Wilen, C., Orchard, R., et al. (2016). Optimized sgRNA design to maximize activity and minimize off-target effects of CRISPR-Cas9. Nat. Biotechnol. *34*, 184–191. https://doi.org/10.1038/nbt.3437.

27. Li, H., and Durbin, R. (2010). Fast and accurate long-read alignment with Burrows-Wheeler transform. Bioinformatics *26*, 589–595. https://doi.org/10.1093/bioinformatics/btp698.

28. Farek, J., Hughes, D., Mansfield, A., Krasheninina, O., Nasser, W., Sedlazeck, F.J., Khan, Z., Venner, E., Metcalf, G., Boerwinkle, E., et al. (2018). xAtlas: scalable small variant calling across heterogeneous next-generation sequencing experiments. Preprint at bioRxiv. https://doi.org/10.1101/295071.

29. Roller, E., Ivakhno, S., Lee, S., Royce, T., and Tanner, S. (2016). Canvas: versatile and scalable detection of copy number variants. Bioinformatics *32*, 2375–2377. https://doi.org/10.1093/bioinformatics/btw163.

30. Corral-Serrano, J.C., Lamers, I.J.C., van Reeuwijk, J., Duijkers, L., Hoogendoorn, A.D.M., Yildirim, A., Argyrou, N., Ruigrok, R.A.A., Letteboer, S.J.F., Butcher, R., et al. (2020). PCARE and WASF3 regulate ciliary F-actin assembly that is required for the initiation of photoreceptor outer segment disk formation. Proc. Natl. Acad. Sci. USA *117*, 9922–9931. https://doi.org/10.1073/pnas.1903125117.

OMTM, Volume *29*

Supplemental information

CRISPR-Cas9 correction of a nonsense mutation in *LCA5* rescues lebercilin expression and localization in human retinal organoids

Tess A.V. Afanasyeva, Dimitra Athanasiou, Pedro R.L. Perdigao, Kae R. Whiting, Lonneke Duijkers, Galuh D.N. Astuti, Jean Bennett, Alejandro Garanto, Jacqueline van der Spuy, Ronald Roepman, Michael E. Cheetham, and Rob W.J. Collin

SUPPLEMENTAL INFORMATION

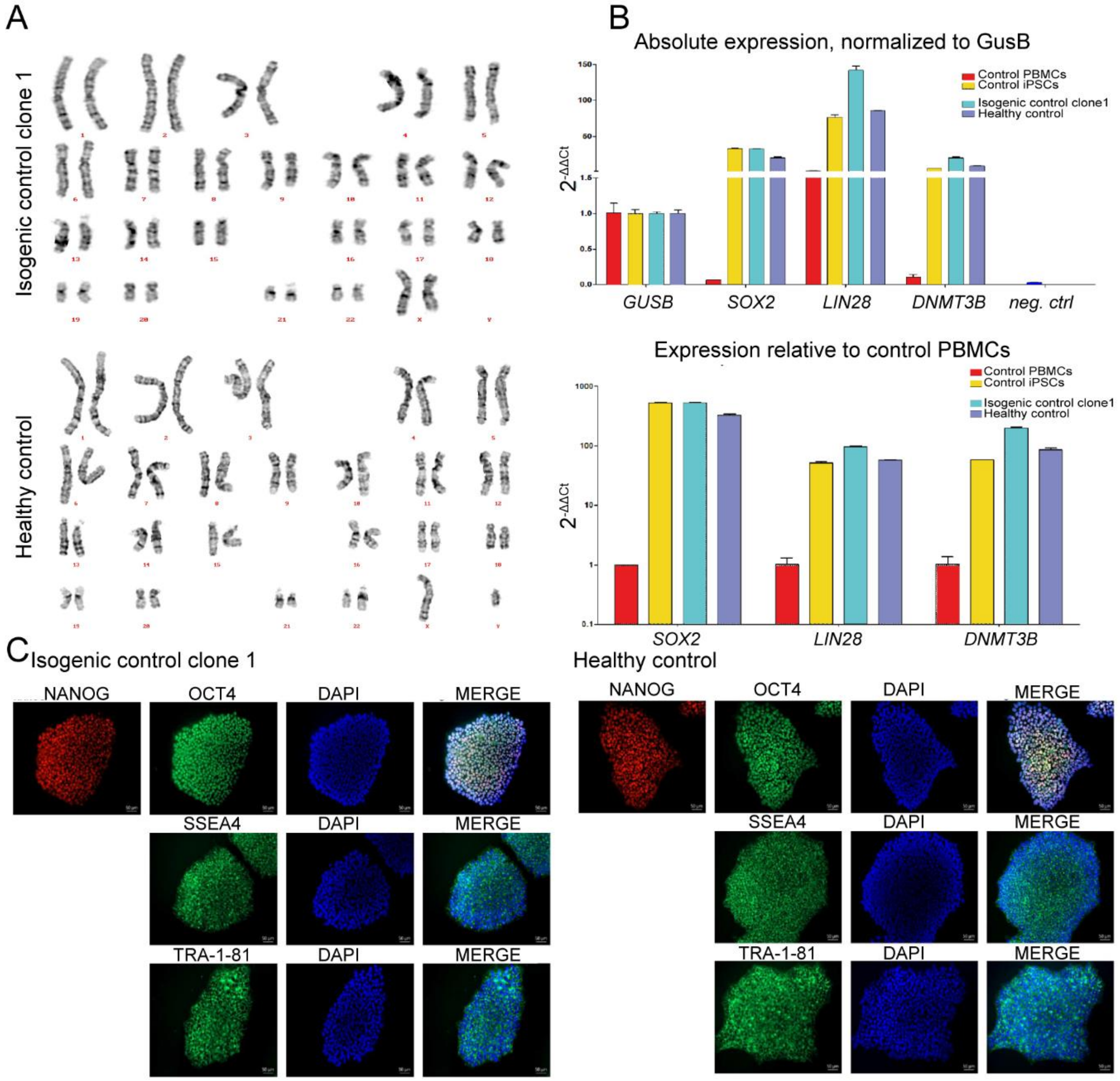

Figure S1. Quality control of healthy control and isogenic control iPSCs. A) Representative karyotypes of isogenic control clone1 and healthy control lines. B) Expression of pluripotency genes SOX2, LIN28, and DNMT3B. C) Immunocytochemical detection of the pluripotency markers OCT4, NANOG, SSEA4, and TRA1-81. Scale bars correspond to 50 µm.

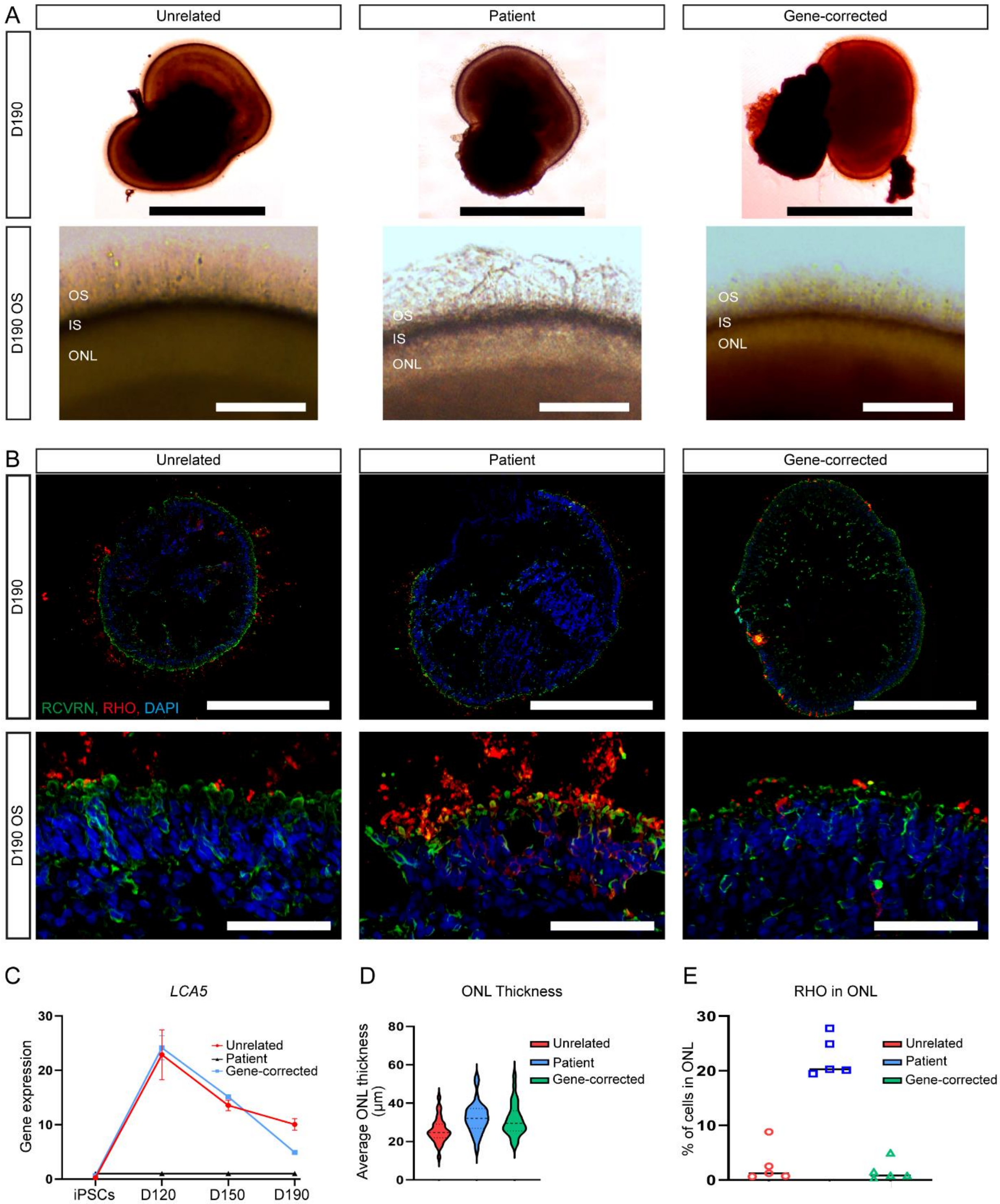

Figure S2. Patient, gene-corrected, and unrelated control human ROs show outer segment-like structures and photoreceptor-cell marker positive cells in the outer nuclear layer. A) D190 ROs generated from LCA5 c.835C>T, gene-corrected and unrelated controls show lamination (top panel, scale bars 1 mm) and brush-like immature outer segments (bottom panel, scale bars 50 µm). B) Representative images of rhodopsin and recoverin-positive outer nuclear layer (ONL) in D190 retinal organoids (top panel scale bars 500 µm and 50 µm, top and bottom panels, respectively). RCVRN

in green, RHO in red, DAPI in blue. C) qPCR of LCA5 expression in iPSCs, D120, D150, and D190 ROs (n=3 ROs per measurement, in duplicate). D) Thickness of ONL among the D190 organoids was 25.8 µm (SD = 6.4 µm), 32.4 µm (SD = 7.9 µm), and 31.2 (SD = 8.4 µm), for unrelated control, patient, and gene-corrected control, respectively (n=30 measurements and N=3 for unrelated control, n=30 measurements and N=1 organoid for the patient, and n=29 measurements and N=2 for isogenic control organoids). E) Rhodopsin signal in the ONL layer of D190 retinal organoids was quantified to be equal to 2.8% (SD = 3.5%), 22.5% (SD = 3.7%), and 1.7% (SD = 1.9%), in unrelated control, patient, and gene-corrected control, respectively (n=5 measurements and N=3 for unrelated control, n=5 measurements and N=1 organoid for patient, and n=5 measurements and N=2 for isogenic control organoids).

Table S1. Primer sequences.

Primer	Primer sequence (5’- 3’)
TIDER primer a	CTCCTGCCTAGGCCTCTCAAAGC
TIDER primer b	TCAACCATGCAACACAGTGAAGCT
Alt-R CRISPR-Cas9 crRNA1	GCGACTATATCACAAATTAA
Template gRNA1	ATATGAATAGTAACATTTAGTGTTTTAAAATATTTAATTTTCGTCTAATACAC<br>ATTATGGAAAACAATGCAATTTAAAATACTTACTTTAAGTTTGTGATATAGTC<br>GCTGTACCTCCTTTTGAAGAA
Template 1 TIDER primer d	GTACAGCGACTATATCACAAACTTAAAGTAAGTATTTTAAATTGC
Template 1 TIDER primer c	TTTAAAATACTTACTTTAAGTTTGTGATATAGTCGCTGTACCTCCTTTTGAAG
Alt-R CRISPR-Cas9 crRNA2	GAAATAAAGTTCTTCAAAAGG
Template gRNA2	AAATATTTAATTTTCGTCTAATACACATTATGGAAAACAATGCAATTTAAAAT<br>ACTTACCTTTAATTTGTGATATAGTCGCTGTACTTCCTTTTGCAAAACTTTATT<br>TTCATCATGAGCCTCATATG
Template 2 TIDER primer d	TTTGCAAAAGGAAGTACAGCGACTATATCAC
Template 2 TIDER primer c	GTGATATAGTCGCTGTACTTCCTTTTGCAAAACTTTATTTTCATC
qPCR primer LCA5_ex3_FWD	AGACACCTACCTGAACGAGATG
qPCR primer LCA5_ex4_REV	CTCAGTTCAAGGTTTTTCGATAGC

Table S2. Antibodies.

Antigen	Species	Dilution	Supplier	Catalog number
Opsin Red/Green	Rabbit	1:500	Millipore	AB5405
Lebercilin	Rabbit	1:100	Home-made	n/a
ARL13B	Mouse	1:250	NeuroMab	75-287
Rhodopsin (4D2)	Mouse	1:500	Novus Biologicals	NBP2-59690
Recoverin	Rabbit	1:500	Abcam	ab5585
